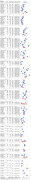# The Prevalence of Neuropsychiatric Symptoms in Alzheimer's Disease: An Updated Systematic Review and Meta‐Analysis

**DOI:** 10.1002/alz70857_106948

**Published:** 2025-12-25

**Authors:** Arkansh Sharma, Oorvi Gupta, Victor Ghosh, Himanshu Arora, Poorvikha Gowda, Marium Khan, Vinay Suresh

**Affiliations:** ^1^ Government Medical College, Omandurar, Chennai, Tamil Nadu, India; ^2^ Lady Hardinge Medical College, New Delhi, New Delhi, India; ^3^ Andhra Medical College, Visakhapatnam, Andhra Pradesh, India; ^4^ Netaji Subhash Chandra Bose Subharti Medical College, Meerut, Uttar Pradesh, India; ^5^ St John's Medical College, Bangalore, Karnataka, India; ^6^ Jinnah Sindh Medical University, Karachi, Sindh, Pakistan; ^7^ King George's Medical University, Lucknow, Uttar Pradesh, India

## Abstract

**Background:**

Neuropsychiatric symptoms (NPS) are integral to Alzheimer's disease (AD) yet often go under‐recognized, despite their profound impact on disease progression, patient outcomes, and care strategies.

**Method:**

A systematic search was conducted in PubMed, EMBASE, SCOPUS, Ovid, and Web of Science up to January 19, 2025. Studies were included if they were original research with a cross‐sectional or longitudinal design, involving patients with probable or possible AD diagnosed according to DSM‐IV or NINCDS‐ADRDA criteria, and reported the prevalence of NPS. The inverse variance method and Freeman‐Tukey double arcsine transformation were used for the proportion analysis. For the odds ratio analysis, the inverse variance method (random effects model) was used with tau^2 estimated by the restricted maximum‐likelihood estimator (RLE) and confidence intervals (CI) via Q‐Profile.

**Result:**

In our meta‐analysis of neuropsychiatric symptoms in Alzheimer's disease (AD), we analyzed 400 studies with 3,189,267 observations and 306,880 events. The individual symptoms in decreasing order of their proportion were: **Apathy** (0.4748 [0.3875; 0.5630]), **Irritability** (0.4120 [0.3612; 0.4636]), **Depression/Dysphoria** (0.3802 [0.3253; 0.4366]), **Agitation** (0.3304 [0.2756; 0.3877]), and **Anxiety** (0.3072 [0.2541; 0.3629]). There was substantial heterogeneity across studies (I^2 = 99.9%). For deriving pooled odds ratios we analyzed 125 studies with a total of 266,938 observations for the odds ratio. None of the individual symptoms had a significant association. **Aberrant motor behavior** exhibited the highest association with AD (OR = 12.90 [0.63, 262.46]), followed by **wandering** (OR = 2.70 [0.06, 128.85]), **psychosis** (OR = 1.81 [0.37, 8.82]), **delusions** (OR = 1.52 [0.67, 3.44]), and **apathy** (OR = 1.33 [0.57, 3.12]). Substantial heterogeneity was observed (*I* ^2^ = 97.4%).

**Conclusion:**

Our meta‐analysis reveals that the most prevalent neuropsychiatric symptoms in Alzheimer's disease are apathy, irritability, and depression/dysphoria, with substantial heterogeneity across studies.